# Machine learning-based suggestion for critical interventions in the management of potentially severe conditioned patients in emergency department triage

**DOI:** 10.1038/s41598-022-14422-4

**Published:** 2022-06-22

**Authors:** Hansol Chang, Jae Yong Yu, Sunyoung Yoon, Taerim Kim, Won Chul Cha

**Affiliations:** 1 Department of Emergency Medicine, Samsung Medical Center, Sungkyunkwan University School of Medicine, 115 Irwon-ro Gangnam-gu, Seoul, 06355 Republic of Korea; 2 Department of Digital Health, Samsung Advanced Institute for Health Science & Technology (SAIHST), Sungkyunkwan University, 115 Irwon-ro Gangnam-gu, Seoul, 06355 Republic of Korea; 3 Digital Innovation Center, Samsung Medical Center, 81 Irwon-ro Gangnam-gu, Seoul, 06351 Republic of Korea

**Keywords:** Diseases, Health care

## Abstract

Providing timely intervention to critically ill patients is a challenging task in emergency departments (ED). Our study aimed to predict early critical interventions (CrIs), which can be used as clinical recommendations. This retrospective observational study was conducted in the ED of a tertiary hospital located in a Korean metropolitan city. Patient who visited ED from January 1, 2016, to December 31, 2018, were included. Need of six CrIs were selected as prediction outcomes, namely, arterial line (A-line) insertion, oxygen therapy, high-flow nasal cannula (HFNC), intubation, Massive Transfusion Protocol (MTP), and inotropes and vasopressor. Extreme gradient boosting (XGBoost) prediction model was built by using only data available at the initial stage of ED. Overall, 137,883 patients were included in the study. The areas under the receiver operating characteristic curve for the prediction of A-line insertion was 0·913, oxygen therapy was 0.909, HFNC was 0.962, intubation was 0.945, MTP was 0.920, and inotropes or vasopressor administration was 0.899 in the XGBoost method. In addition, an increase in the need for CrIs was associated with worse ED outcomes. The CrIs model was integrated into the study site's electronic medical record and could be used to suggest early interventions for emergency physicians.

## Introduction

Clinical decision support systems based on artificial intelligence (AI)-related algorithms are evolving rapidly^1^. As of 2020, the Food and Drug Administration has approved several AI-based algorithms in diverse medical fields^2^. These algorithms are especially used to support the analysis of test results, such as imaging interpretations. While clinical AI algorithms have also been developed, relatively few provide support for decision-making.

AI has particular potential in the emergency department (ED) owing to the need for proper distribution of resources and accurate decision-making^3^. Analyzing clinical data and predicting condition severity are needed for effective ED operation, for which AI may be applied to improve clinical prediction^4–6^. Several studies have introduced AI-based triage systems and methods for predicting disease outcomes^5,7,8^. Considering the time sensitivity and necessity for emergency care in the ED, the role of these AI-based decision support systems might expand.

In the ED, timely and appropriate interventions as well as adequate screening of the severity of a patient’s condition are important in critically ill patients^9–12^, especially as these patients need intensive care^13^. ED resources, however, are limited. Therefore, rapid screening and timely, appropriate, and focused interventions are vital for critical patients in the ED environment, especially when it is crowded and delayed recognition and treatment of critical patients can result in mortality^14,15^.

However, previously reported models of predicted outcomes or clinical course prediction such as ICU admission or mortality and did not guide the deductive process for critical patients, such as the exact procedures or prescription of medications^16–20^. Thus, decisions regarding intervention are made entirely by the clinician’s judgment based on his or her experiences. Clinical result prediction might be insufficient to improve the clinical outcome, because action should be required. For instance, without the proper administration of inotropes or transfusion in a hemorrhagic shock patient, the patient will deteriorate and will be unable to compensate^21–23^. However, early and proper management and transfusion may help stabilize the patient and allow for further evaluation and management and finally might be able to improve outcome. Outcome prediction might be helpful for predicting patient course, but does not support the exact decision of action for care. The actual management action itself, inotropes or transfusion improved patient status.

Additionally, clinicians may experience difficulty making decisions in a timely ED environment, such as crowding or a lack of resources, and experience^24,25^. Predicting outcomes alone may not be sufficient; decision on clinical procedures and actions should be made. Therefore, in this study, we aimed to predict the need for a suggestion of actual interventions in patients with critical conditions. Therefore, in this study, we aimed to predict the need for a suggestion of actual interventions in patients with critical conditions.

## Results

Between 2016 and 2018, 223,585 patients have visited study site ED. Among them, Out-hospital Cardiac Arrest, Dead on arrival, patient without record of initial treatment, left without being Seen, patient without Korean Triage and Acuity Scale (KTAS), Injured patient and age under 18 was excluded. A total of 137,883 patients met the pre-specified criteria and were included in the final analysis (Supplementary Fig. 1). Among these patients, 9.3% (12,922) received at least one of critical interventions (CrIs). The percentage of the total population and its corresponding number of patients that received CrIs are as follows: 1.0% (1355) for arterial line (A-line) insertion, 7.6% (10,577) for oxygen therapy, 0.42% (581) for high-flow nasal cannula (HFNC), 0.64% (878) for intubation, 0.05% (66) for massive transfusion protocol (MTP), and 2.52% (3471) for inotropes or vasopressor administration.

Almost all characteristics differed between critical care and non-critical care patients. The median patient ages were 67.0 and 57.0 years, respectively. Composite adverse outcomes were more likely to occur in patients who receives CrIs (25.4% and 1.6%, respectively). The demographic characteristics are summarized in Table 1. Number of missing data and frequecy of each variable are presented in the Supplemental Table 1.

**Table 1:** Basic characteristics of the study population between patients who received at least one critical intervention and those who did not receive any intervention.

	^a^At least one CrIs (*n* = 12,922)	Without CrIs (*n* = 124,911)	*p*-value^a^
Age, median [IQR]	67.0 [57.0, 77.0]	57.0 [42.0, 68.0]	< 0.001
**Sex, n (%)**			< 0.001
Male	7659 (59.3%)	59,893 (47.9%)	
Female	5263 (40.7%)	65,018 (52.1%)	
SBP (mmHg), median [IQR]	119.0 [98.0, 141.0]	128.0 [112.0, 145.0]	< 0.001
DBP (mmHg), median [IQR]	69.0 [56.0, 82.0]	77.0 [67.0, 87.0]	< 0.001
PR (beats/min), median [IQR]	102.0 [85.0, 118.0]	86.0 [75.0, 100.0]	< 0.001
RR (breaths/min), median [IQR]	20.0 [18.0, 22.0]	18.0 [18.0, 20.0]	< 0.001
Temperature (°C), median [IQR]	37.1 [36.6, 37.7]	36.8 [36.5, 37.3]	< 0.001
SpO_2_ (%), median [IQR]	96.0 [93.0, 98.0]	98.0 [97.0, 99.0]	< 0.001
**KTAS, n (%)**			< 0.001
1	1044 (8.1%)	336 (0.3%)	
2	2964 (22.9%)	8436 (6.8%)	
3	7364 (57.0%)	58,777 (47.1%)	
4	1400 (10.8%)	48,118 (38.5%)	
5	150 (1.2%)	9244 (7.4%)	
**Mental status, n (%)**			< 0.001
Alert	10,900 (84.4%)	122,895 (98.4%)	
Verbal	723 (5.6%)	1285 (1.0%)	
Pain	696 (5.4%)	627 (0.5%)	
Unresponsive	603 (4.7%)	104 (0.1%)	
**Mode of arrival, n (%)**			< 0.001
Ambulance	6886 (53.3%)	21,241 (17.0%)	
Other	6036 (46.7%)	103,670 (83.0%)	
**Discharge, n (%)**			< 0.001
Home	2593 (20.1%)	89,905 (72.0%)	
ED death	569 (4.4%)	28 (0.0%)	
Transfer	1171 (9.1%)	2706 (2.2%)	
Admission	8589 (66.5%)	32,272 (25.8%)	
**Admission, n (%)**			< 0.001
ICU	2717 (31.6%)	1964 (6.1%)	
GW	5872 (68.4%)	30,308 (93.9%)	
Composite adverse outcome	3,286 (25.4%)	1992 (1.6%)	< 0.001

*CrIs* critical interventions, *SD* standard deviation, *SBP* systolic blood pressure, *DBP* diastolic blood pressure, *PR* pulse rate, *RR* respiratory rate, *TEMP* temperature, *SpO*_*2*_ peripheral capillary oxygen saturation, *KTAS* Korean Triage Acute Scale, *GW* general ward, *ED* emergency department, *ICU* intensive care unit, *Composite Adverse Outcome* ICU admission or ED death.

^a^*p-*values were calculated using independent t-tests for continuous variables and chi-square tests for categorical variables.

Supplementary Fig. 2 shows time differences between emergency room visits and each CrIs. The median hours of each CrIs from ER visit were 3.44, 1.91, 5.70, 1.60, 2.92, and 3.78 for A-line insertion, inotropes, HFNC, intubation, MTP, and oxygen therapy, respectively.

Table 2 summarizes the area under the receiver operating curve (AUROC) and area under the precision-recall curve (AUPRC) for each CrIs using the evaluation method with 95% Confidential intervals (CI). The AUROC of predictions of need was 0.913, 0.909, 0.962, 0.945, 0.920, and 0.899 for A-line insertion, oxygen therapy, HFNC, intubation, MTP, and inotropes, respectively. The AUPRC was very different for each CrIs, owing to differences in their prevalence. The AUROC and AUPRC results for other input factors are depicted in Supplementary Table 1, which illustrates the prediction results of AUROC and AUPRC with 95% CI according to the machine learning methods.

**Table 2:** Results of prediction need of CrIs AUROC and AUPRC with 95% CIs according to the machine learning method (XGBoost).

	AUROC (CI)	AUPRC (CI)	Sen (95% CI)	Spec (95% CI)	PPV (95% CI)	NPV (95% CI)
A-line	0.913 (0.899–0.927)	0.121 (0.112–0.130)	0.866 (0.822–0.907)	0.821 (0.785–0.854)	0.042 (0.035–0.049)	0.998 (0.998–0.999)
Oxygen therapy	0.909 (0.904–0.916)	0.576 (0.570–0.583)	0.812 (0.780–0.846)	0.853 (0.819–0.881)	0.313 (0.275–0.353)	0.982 (0.979–0.985)
HFNC	0.962 (0.948–0.976)	0.207 (0.189–0.230)	0.922 (0.873–0.964)	0.906 (0.865–0.941)	0.043 (0.029–0.061)	0.999 (0.999–0.999)
Intubation	0.945 (0.932–0.958)	0.193 (0.180–0.203)	0.891 (0.817–0.940)	0.865 (0.818–0.946)	0.047 (0.032–0.091)	0.999 (0.998–0.999)
MTP	0.920 (0.849–0.991)	0.014 (0.011–0.018)	0.878 (0.722–1.00)	0.896 (0.871–0.982)	0.005 (0.002–0.015)	0.999 (0.999–1.00)
Inotropics and vasopressors	0.899 (0.888–0.911)	0.388 (0.379–0.399)	0.826 (0.783–0.863)	0.827 (0.788–0.868)	0.104 (0.08–0.125)	0.995 (0.993–0.996)

Cut-off value of prediction model for calculating Spec, Sen, PPV, NPV was set by youden index. *XGBoost* extreme gradient boosting, *HFNC* high-flow nasal cannula, *MTP* massive transfusion, *AUROC* area under the receiver operating characteristic curve, *AUPRC* area under the precision-recall curve, *CI* confidence interval, *Sen* Sensitivity, *Spec* Specificity, *PPV* Positive predict value, *NPV* Negative Predict value.

**Figure 1:**
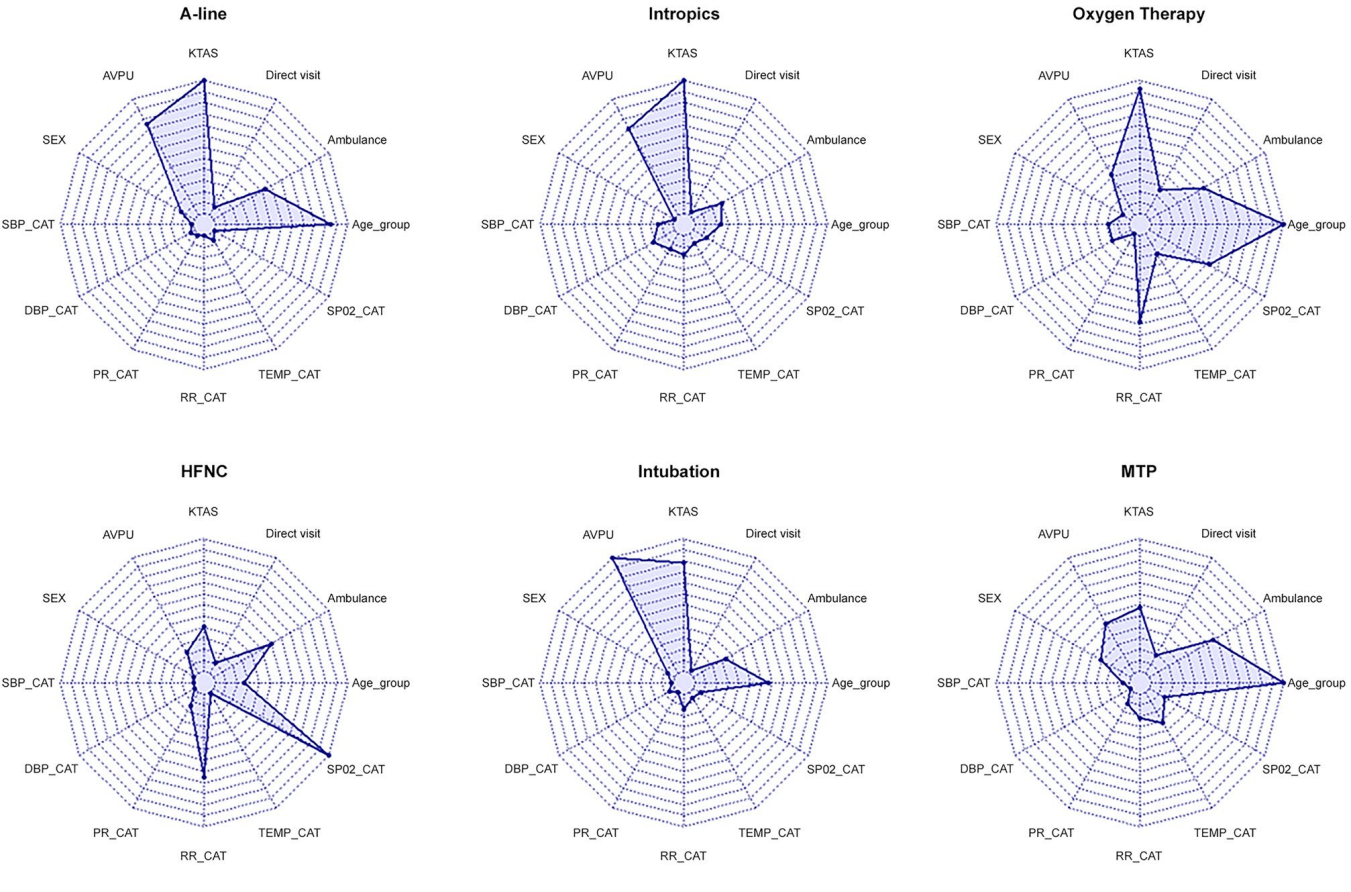
Feature importance of the input factors for the random forest plot of predictive variables for each critical intervention. These included demographic information such as age group and sex and initial nursing assessment information such as categorized vital signs and severity.

Figure 1 shows the feature importance of each CrIs. Age group and KTAS were the most impactful for A-line insertion, oxygen therapy, and intubation, while vital signs such as Respiratory Rate (RR) and Oxygen Saturation (SpO_2)_ were the most relevant for HFNC and MTP.

According to the model based on cut-offs determined by the Youden index, the sensitivities were 0.85, 0.81, 0.88, 0.90, 0.99, and 0.84 and the specificities were 0.75, 0.85, 0.88, 0.86, 0.86, and 0.82 for A-line insertion, oxygen therapy, HFNC, intubation, MTP, and administration of inotropes and vasopressors, respectively. Other details are shown in.

Supplementary Fig. 3 illustrates the well-calibrated relationship between observed and predicted results for each CRIs model, except for the MTP. MTP also displayed a similar trend, despite the fact that it had a greater degree of variation when compared to other procedures, owing to its low prevalence.

Supplementary Table 2, which illustrates the prediction results of AUROC and AUPRC with 95% CI according to other input factors.

Table 3 shows the odd ratios (OR) of adverse outcomes in the ED according to the number of predicted CrIs. Compared to patients who were predicted to have 0 CrIs, that of patients who were predicted to have one CrIs was 3.10, that of two CrIs was 5.40, that of three CrIs were 6.83, that of four CrIs was 11.23, that of six CrIs was 23.05, and that of six CrIs was 33.43. As the predicted number of CrIs increased, the OR of adverse outcomes also increased.

**Table 3:** Adjusted risk factors for the number of critical interventions according to clinical outcome, defined as in-hospital mortality or intensive care unit admission.

Number of CrIs	*n*	Risk factor analysis		
		Odds ratio	95% CI	*p*-value
0	27,668	1 (ref)		
1	4364	3.10	2.51–3.82	< 0.001
2	1904	5.40	4.28–6 83	< 0.001
3	2082	6.83	5.53–8.43	< 0.001
4	1535	11.23	9.16–13.75	< 0.001
5	3122	23.05	19.88–26.74	< 0.001
6	675	33.43	27.10–41.23	< 0.001

CrI Odds ratios were adjusted for age group, sex, KTAS, AVPU, route of ER visit, method of transportation, and categorized vital signs in phase.

**Figure 2:**
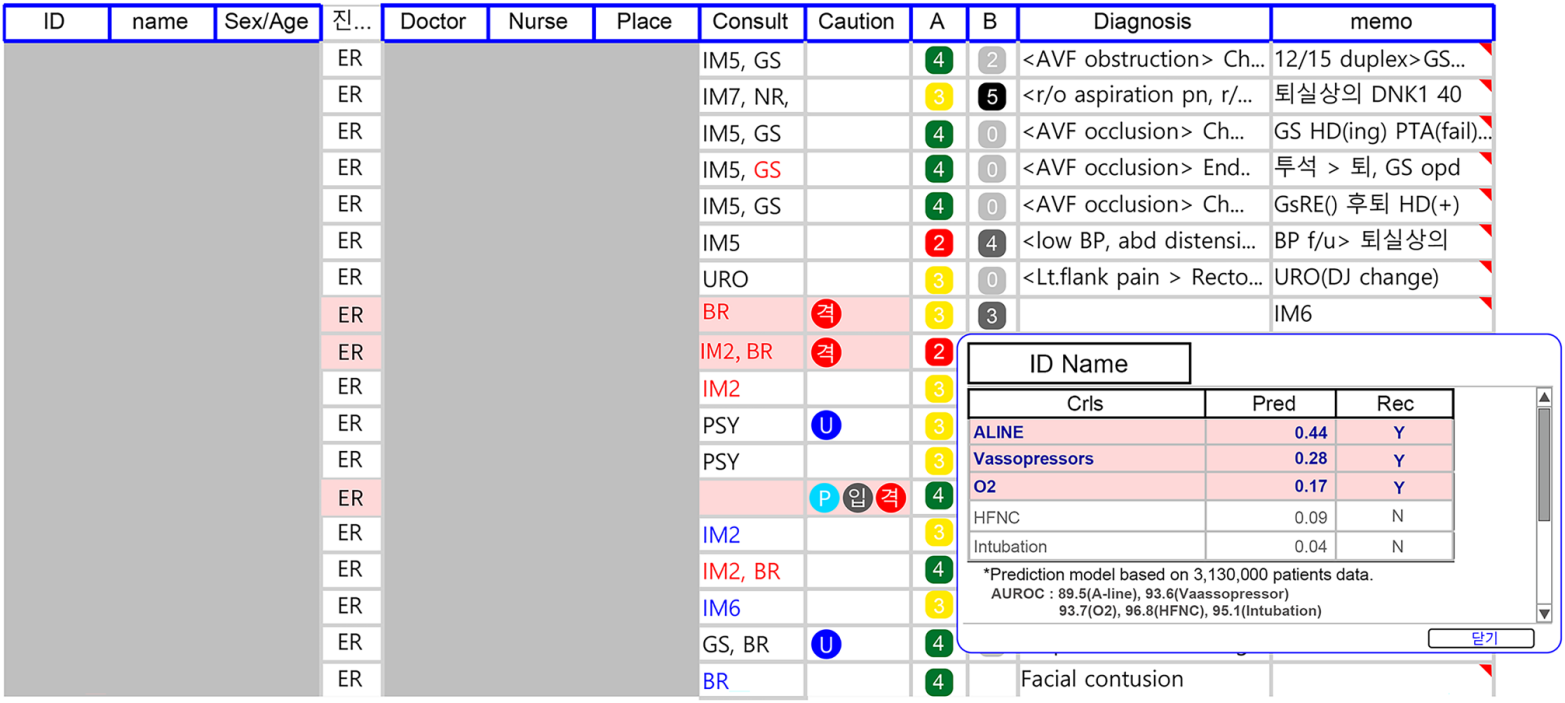
Integration of CrIs into the study site's electronic medical record. We developed the CrIs model in the study site's electronic medical record. The threshold was determined using the Youden index. (**A**) The Korean Triage Acuity Score; (**B**), the Critical Intervention Score, indicates the number of Critical Interventions that are predicted to be required. (**C**) A box containing the exact prediction value and recommendation appears when the number icon in the CrIs column is clicked.

The CrIs model was integrated into the electronic medical record at the study site and used to determine the need for each intervention (Fig. 2). The number of CrIs was displayed alongside the KTAS. Additionally, a pop-up window indicates which interventions should be considered and their associated properties during a patient's initial visit to the ED. The Youden index was used to determine the recommended threshold for each model.

## Discussion

This study varies from earlier research in that CrIs were defined as the predictive outcome utilizing only triage variables that may be collected immediately after a patient enters the emergency department (ED) without requiring laboratory results and still shows a high AUROC. In addition, this prediction result demonstrated that the number of predicted CrIs was associated with adverse patient outcomes in ED. Intensive care unit (ICU) admission or ED death increases in patient with at least one CrIs done (Table 1). Table 3 shows that the number of predicted CrIs was associated with ICU admission and in-hospital mortality, even after adjusting for other variables. As critical patients generally require interventions, our finding suggests that CrIs also might be used to assess or predict the severity of patients' conditions in the emergency department.

This study did not focus on predicting adverse outcomes, but instead focused on prompt interventions, which can support major clinical decisions and potentially improve patients’ outcomes. Recent review in literatures demonstrated that major predictors for AI are patient's conditions as recognizing clinical conditions, predicting disease evolution, or predicting outcome^26–28^. In recent years, several AI-related studies have concentrated on clinical decision-making, action-based learning, such as using reinforcement learning^26,29^. This study suggests actions, not only decisions, in which it is distinct from those of earlier research^30–33^.

Furthermore, by suggesting interventions directly and separately, this model may support clinician decision and action more in specific way. For less experienced clinicians or those working in crowded emergency departments, clinician decision-making and prioritization are difficult to be in good quality^24,25^. These suggestions might help reassure clinicians about initiating interventions, identifying necessary interventions that are missed, or reconsidering interventions. These critical interventions should be performed selectively and carefully because delays can be harmful and result in deterioration of patient or unnecessary procedures can result in wastage of ED resources, may harm patients, and may delay the proper treatment of other patients due to limited ED resources^30–33^. Prediction result might support clinical decision and allowing the selective and efficient use of resources. In future study, suggestions for these actions by AI should also be compared to those by physicians.

The results of this study may also help guide clinicians by prediction making before test results are available, as the variables used in this study can be obtained in the ED to allow early prediction. This finding is also supported by the fact that most CrIs were performed in the initial stage of ED stay. In most cases of critical patient care in the ED, appropriate and timely treatment is important. Under or delayed treatment can lead to high mortality, especially in critical patient care^34–36^. Therefore, our prediction model can help resuscitate critically ill patients.

Finally, this model was integrated into the real world as Fig. 2. The number of CrIs indicates the anticipated number of interventions and can be used as a secondary severity scaling tool. Additionally, the new integrated CrIs model identifies which procedures should be considered during a patient's initial visit to the ED and makes specific recommendations regarding specific interventional actions. Additional validation and monitoring user’s opinion is needed in future.

However, there are some limitations in our study. First, the study was a single-center retrospective study, and thus, may have had a selection bias. Therefore, caution is required when applying these findings to other hospitals. Second, six CrIs that are well known to be related to resuscitation and critical care were selected. However, no guidelines indicate the interventions that are needed in critically ill patients and when these interventions should be performed. A consensus is needed to standardize the CrIs that are required in critical care and when they should be performed. Third, incidence of six CrIs is modest and imbalanced. As a result, all CrIs predictions had a low AUPRC and positive predict value. The majority of candidates have a low likelihood and incidence of outcome in the medical area because the majority of medical outcomes, including CrIs, occur in an unhealthy state. As this is a study based on real-world data, this imbalance may be a constraint. Rather than that, our model exhibits a high degree of specificity and sensitivity. Several more machine-learning-based prediction studies in the medical profession make the similar point^37^. It is important for users to understand the limitation and bias of AI algorithms in real practical settings. Finally, this study does not consider patients who are seriously ill and receiving palliative care. Because they may require life-sustaining treatments, they may refuse life-sustaining care or critical interventions. These individuals may become bias of the secondary outcome survey because they were admitted to the general ward for palliative care rather than the intensive care unit, despite their severe condition and need for interventions. Users of the system, such as emergency physicians should be aware of the potential bias of the algorithm.

This study suggests a new point of view regarding the prediction of required care and interventions for critical patients by predicting the need for specific intervention in the early stage of the ED stay rather than predicting the endpoint of the ED process.

## Methods

We built models to predict interventions performed in the ED, including A-line insertion, oxygen therapy, HFNC, intubation, massive transfusion protocol MTP, and administration of inotropes and vasopressors.

This study was approved by the Institutional Review Board (IRB) of the Samsung Medical Center (IRB No. 2021-03-132). The need for informed consent was waived by the institutional Review Board (IRB) of the Samsung Medical Center Samsung Medical Center because of the retrospective, observational, and anonymous nature of the study. All methods were performed in accordance with the relevant guidelines and regulations. It was not appropriate or possible to involve patients or the public in the design, or conduct, or reporting, or dissemination plans of our research public were not involved in the design, or conduct, or reporting, or dissemination plans of this research.

### Study setting and population

This retrospective study was conducted in the ED of a tertiary hospital located in a metropolitan city. The hospital has approximately 1960 inpatient beds. The annual number of patients in the ED was approximately 80,000.

We included patients who visited the ED between January 1, 2016, and December 31, 2018 and excluded those who visited without receiving treatment or who left without being seen by a clinician, those with injury, those aged < 18 years, and those who were dead on arrival (DOA); patients who were DOA were excluded because there was no need to predict the clinical outcomes. Out Hospital Cardiac arrest patients were also excluded because they present ED as arrest state, and need immediate intervention, including advanced cardiac life support, and there is no need for clinical support for the prediction.

### Primary outcome

The primary outcomes of this study included several critical interventions (CrIs) that are performed in the ED of the study center, which are closely related to the resuscitation of critical patients, including Arterial line insertion, oxygen therapy, High flow nasal cannula (HFNC), intubation, Massive transfusion protocol (MTP), and administration of inotropes and vasopressors^38,39^. Selected interventions are closely related to the airway, controlling the work of breathing, (intubation), circulation, (A-line, inotropes and vasopressors, Massive Transfusion), adequate oxygen delivery (Oxygen therapy, High flow nasal canula, intubation), and achieving the endpoint of resuscitation (Arterial line monitoring), which is closely related to cardiopulmonary resuscitation^39,40^.

An A-line is needed for arterial blood pressure (ABP) monitoring; Oxygen therapy delivers oxygen patients who has oxygen demand; HFNC is used for oxygen delivery of > 15 L per minute (L/min); intubation for securing the airway or applying mechanical ventilation; and MTP for large volume transfusions, including the administration of six pack each of red blood cells, single donor platelets, and fresh frozen plasma with a level-1 device. Inotropes and vasopressors are administered for cardiopulmonary resuscitation and blood pressure maintenance during shock. Each resuscitation intervention is required for different purposes^41,42^.

### Secondary outcomes

The secondary outcome was defined as mortality in the ED or ICU admission. We analyzed the association between the value of CrIs prediction and clinical results in the ED.

### Predictors selection

We built a prediction and risk factor model based on data from 137,883 patients who visited the ED between January 1, 2016, and December 31, 2018. Triage data, which can be obtained shortly after a patient arrives, was used to predict the need for CrIs early on. Vital information such as body temperature, systolic blood pressure, diastolic blood pressure, respiratory rate, pulse rate, and oxygen saturation, obtained in the triage stage, was used as a predictor. In addition, the mode of arrival, KTAS, and mental status measured initially, which was mandatory when patients visited the ED, were used to build the model.

The mode of arrival refers to the type of transportation used to come to the hospital, for example, via ambulance or on foot. KTAS is a five-level triage system based on the patient’s chief complaint and symptom severity. It is utilized by a trained nurse or doctor during triage. It is used nationwide and has mandatory value in the emergency room in Korea.

We selected only information obtained during the initial triage as mentioned previously, and therefore excluded laboratory test and imaging test results. Vital signs were monitored after entering the ED was also excluded. This is because our study aimed to predict the need for CrIs in the initial stage of ED and laboratory or other tests take time to provide results (Supplementary Fig. 3).

### Data collection

Variables derived from information available in the initial ED triage were included in the development of CrI model. This data was gathered during the initial triage assessment by a nurse. In initial triage, patient information is gathered in accordance with the National Emergency Department Information System (NEDIS), which includes Patient data included demographic information such as age and sexual orientation, as well as information about the ED visit, such as systolic and diastolic blood pressure, pulse rate, respiratory rate, body temperature at arrival, oxygen saturation, KTAS, mental status, and mode of arrival.

Information about whether CrIs were performed on each patient in the ED was also collected. Information about A-line insertion, oxygen therapy, HFNC, and intubation was obtained using a flow chart (Supplementary Fig. 4), which was recorded in real-time and integrated into the electronic medical record system of the study center. Information on MTP was obtained via cross-matching of the prescription code of red blood cells and level-1 device, which is used for massive transfusion. Prescription codes were used to obtain information about the administration of inotropes and vasopressors, including epinephrine, norepinephrine, dopamine, dobutamine, and vasopressin.

We collected patient information from the clinical data warehouse of the study site.

### Data preprocessing

Some continuous variables were transformed into categorical variables for ease of interpretation and nonlinearity. Ages were grouped into 18–40 years, 40–60 years, 60–80 years, and others. The mode of arrival was categorized as public ambulance, private ambulance, and others. The route of arrival was created by direct admission, transfer, and others. Vital signs were categorized as either normal (systolic blood pressure, 100–150 mmHg; diastolic blood pressure, 60–90 mmHg; pulse rate, 50–100 bpm; RR, 12–20 breaths/min; body temperature, 36–37.5 °C; SpO2, 95%–100%) or abnormal if these values were not in the normal range^43,44^. KTAS was used as the original five-level category. Mental status information was collected and divided into four categories: AVPU levels were categorized into alertness, responsive to verbal stimuli, responsive to pain stimuli, and unresponsive. Information about the mode of arrival was divided into two categories: patients who arrived in an ambulance and those who did not.

Information about each CrIs was divided into binary categories whether done or not and processed separately by each of the six CrIs. Time information about when CrIs was performed was also processed separately.

Information about the results after ED stay was categorized as death, ICU admission, general ward (GW) admission, transfer to another hospital, and return to home.

### Missing data handling

As mentioned previously, patient data is collected in accordance with NEDIS system rules. As some features are mandatory field for the NEDIS system, it must be filled in. However, the patient who did not underwent medical care, such as left without being seen, came for medical cortication or was deleted due to registration failure, can have missing vital value, was initially excluded. Except this situation, vital sign recording is mandatory at the study site, and all patients including minor trauma patients should have them completed. Only when it is very urgent situation, and need urgent entrance in emergency room, triage nurse can enter vital sign as ‘uncheckable’. Therefore, we classified ‘uncheckable’ as abnormal. If it is very urgent situation and patient who should be enter ED immediately, triage nurse can enter vital sign as uncheckable. On the other case, if value of vital sign is too low or too high and is out of range it can also entered as ‘uncheckable’. Therefore, we classified ‘uncheckable’ as abnormal because in the study center, patients with high acuities, such as impending arrest or respiratory failure, usually skip the full vital sign measurement in the triage for immediate entrance in the ED and urgent care.

### Model development

The prediction model of CrIs was developed by Extreme Gradient Boosting (XGBoost) and the risk factor model was made with multiple logistic regression based on data from 137,883 patients who visited the ED between January 1, 2016, and December 31, 2018.

We chose the XGBoost algorithm because it is a fast and scalable machine learning algorithm. Several reasons led us to choose the XGBoost algorithm: it is a fast and scalable machine learning technique for gradient boosting mechanisms based on multiple decision trees. First, XGBoost 's benefits have been validated in previous studies^45^. Second, It used the ensemble method, in which new trees are focused on the error from previous tree models^46,47^. Third, the XGBoost algorithm can handle nonlinear interactions between input and output variables^48^. Finally, XGBoost can avoid overfitting by including a regularization term in its objective function^48^.

We built six separate and independent models for each CrIs, not a six-class prediction model, because each CrIs has a different use, and may be used together for same patient to treat specific condition. For example, in hemorrhagic shock patient, A-line will be used for monitoring, while MTP also can used for fast transfusion. However, these models shared the same input variables as model features. Additional feature selection was not made because we had already selected very few predictor candidates initially. The study aimed to predict the need for CrIs in the initial stage, when little information is available.

We divided patients into time periods for model development and validation. The model was developed using data from patients who visited the ED between 2016 and 2017. Validation of the model was conducted using data from patients who visited the ED in 2018.A random search was performed using tenfold cross-validation to obtain the best hyperparameters for each CrIs. To optimize the performance, we considered the maximum tree depth, the minimum amount of data in a node, gamma, subsampling ratio, number of predictors, and learning rate. The considered and selected hyperparameters are listed in Supplementary Table 3.

Moreover, we also implemented other traditional machine learning methods for comparison. Additionally, we performed L2 regularized logistic regression, random forest, and essential ANN (three layers) with the default setting.

The software implemented for model development and validation were R (version 4.1.0), Python programming language (version 3.8.5), Tensorflow framework (version 2.3.1), and scikit-learn (version 0.23.2).

We calculated the AUROC and AUPRC validation datasets. To obtain 95% CI, we implemented 1000 bootstraps for each metric. We also used feature importance radiation plots in a random forest to determine the variables affecting the performance of each CrIs. For practical application, the cut-off for each CrIs model was determined based on the trade-off between sensitivity, specificity, and other possible resources. Candidate cut-offs were calculated with the highest AUROC according to the Youden index, which was the maximum value of the average sensitivity and specificity^49^. Based on this cut-off, false-positive and false-negative findings were identified for each CrIs model.

The calibration plot, which shows how well the prediction probabilities from each model were calibrated, was visualized for the identification of agreement between the predictions and observed outcomes.

### Sensitivity analysis

We performed the same analysis with other input factors for the sensitivity analysis. We considered two other types of input, including the presence of the laboratory order and use of laboratory test results, as follows:

(A) Variables derived from information available in the initial ED triage
(B) A) + presence of lab order

### Model integration

The CrIs model was integrated into the electronic medical record (EMR) at the study site. The hospital’s home-grown EMR, DARWIN refers ‘data analytics and research window for integrated knowledge’. Initial triage inputs were collected in the DARWIN and transferred every 10 min to the interim database (HANA DB), which houses the CRIs models. Finally, each probability was computed and integrated into the DARWIN system (Supplementary Fig. 5).

### Statistical analyses

Descriptive statistics were used for the demographic features and characteristics of ED visits. Categorical variables are expressed as counts and percentages of the total number of data available within the database, and continuous variables are expressed as means and standard deviations. Comparison tests were performed using t-tests and chi-square tests at the 5% significance level. Statistical analysis for inferring the composite outcome of odds ratio (OR) was conducted using multiple logistic regression with a logit link function. We selected variables that showed significance in the univariate logistic regression at a 5% level of significance.

For each CrI, we identified the time difference of ED visits and compared the time of action. In addition, we analyzed the association between the results of CrIs prediction and ED adverse outcomes by calculating the OR.

## Supplementary Information

Supplementary Information.

## Data Availability

Data was available in study site clinical data warehouse. The datasets generated and analyzed during the current study are not publicly available due dataset includes although is de-identified, part of patient information, but are available from the corresponding author on reasonable request.

## Abbreviations

ABP: Arterial blood pressure
AI: Artificial intelligence
A-line: Arterial line
AUROC: Area under the receiver operating curve
AUPRC: Area under the precision-recall curve
CI: Confidence intervals
CrIs: Critical interventions
DOA: Dead on arrival
ED: Emergency department
HFNC: High-flow nasal cannula
ICU: Intensive care unit
IRB: Institutional review board
KTAS: Korean triage and acuity scale
MTP: Massive transfusion protocol
OR: Odds ratio
RR: Respiratory rate
SpO_2_: Oxygen saturation
XGBoost: Extreme gradient boosting
