# Supplementary material for: Machine learning-based suggestion for critical interventions in the management of potentially severe conditioned patients in emergency department triage
Source: Sci Rep. 2022 Jun 22;12:10537. doi: 10.1038/s41598-022-14422-4 (PMC9218081; doi:10.1038/s41598-022-14422-4)
Supplement: Supplementary file 1 — Supplementary Information. [file 41598_2022_14422_MOESM1_ESM.pdf]

Supplementary Figure. 1 Flow chart.

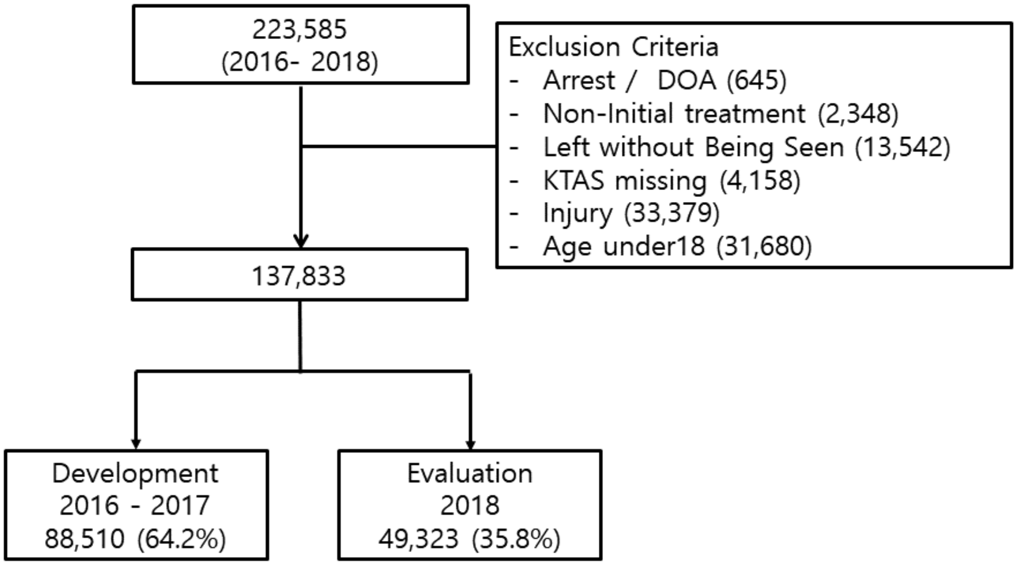

Abbreviation: *Arrest* Out-hospital Cardiac Arrest, *DOA* Dead on arrival, *Non initial treatment* patient without record of initial treatment, *KTAS missing* patient without KTAS, *Injury* Injured patient.

Supplementary Figure. 2 Time differences (in hours) during emergency room visits for each type of critical interventions.

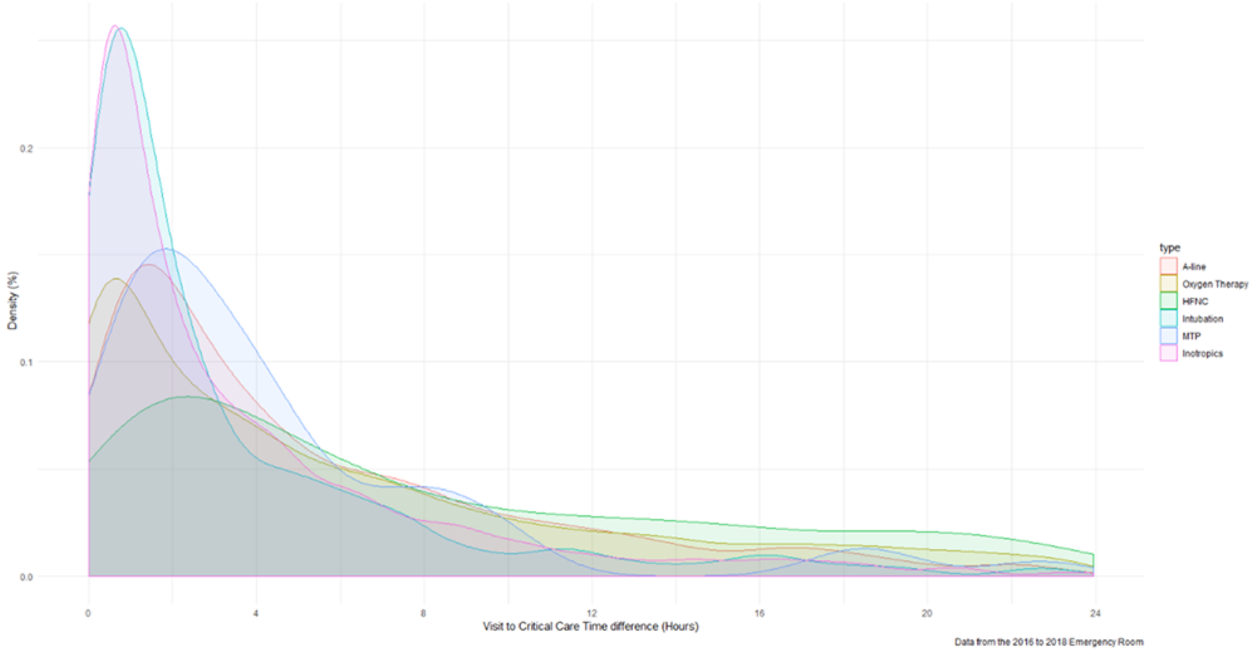

Abbreviation: *A-line* arterial line, *HFNC* high-flow nasal cannula, *MTP* massive transfusion protocol, *Inotropics* inotropic agents including epinephrine, norepinephrine, dopamine, dobutamine, and vasopressin

Supplementary Figure 3. Calibration plot for each CRIs

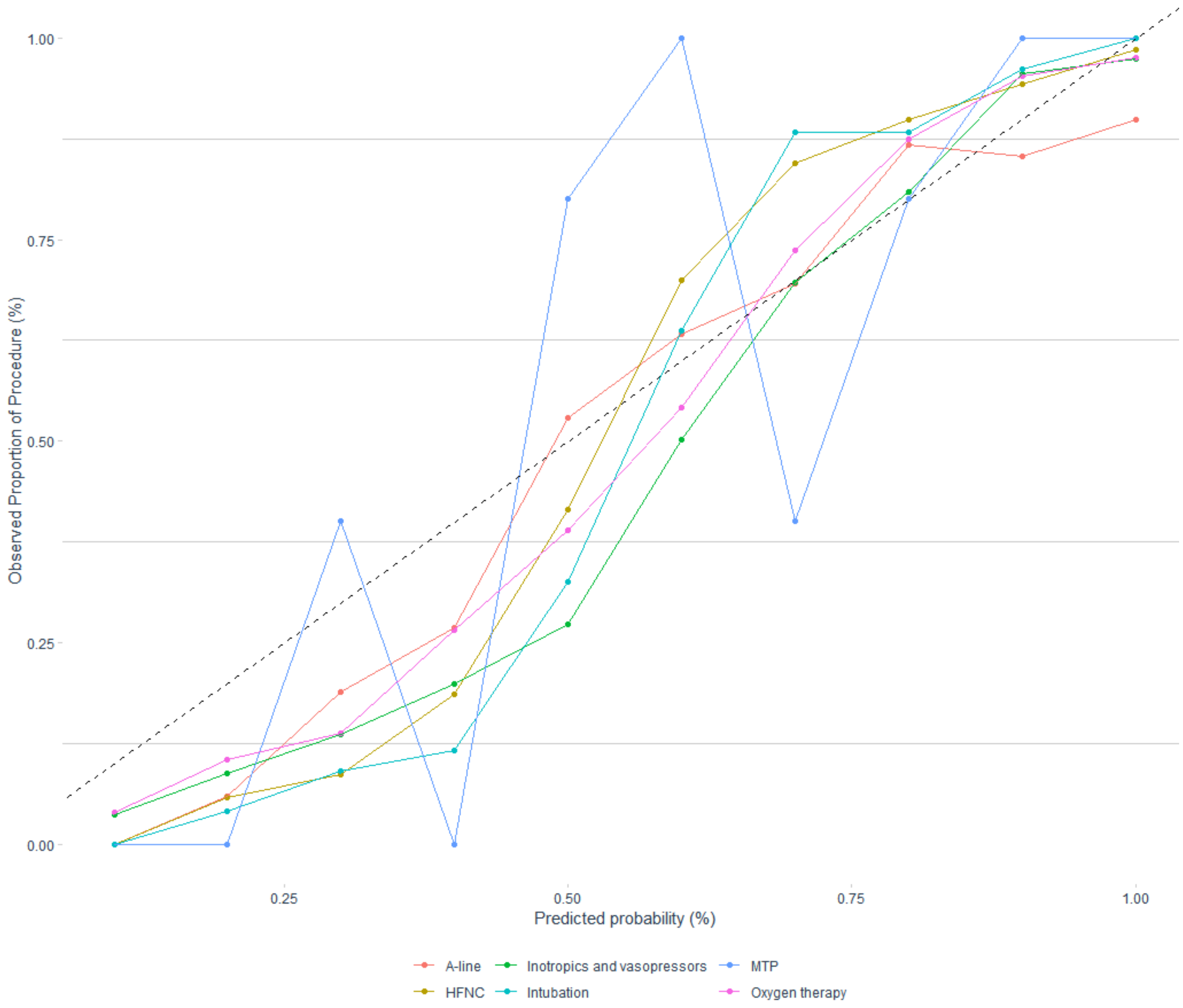

A-line : Arterial line ; MTP : Massive Transfusion Protocol ; HFNC : High Flow Nasal Cannula

Supplementary Figure. 3 Model design.

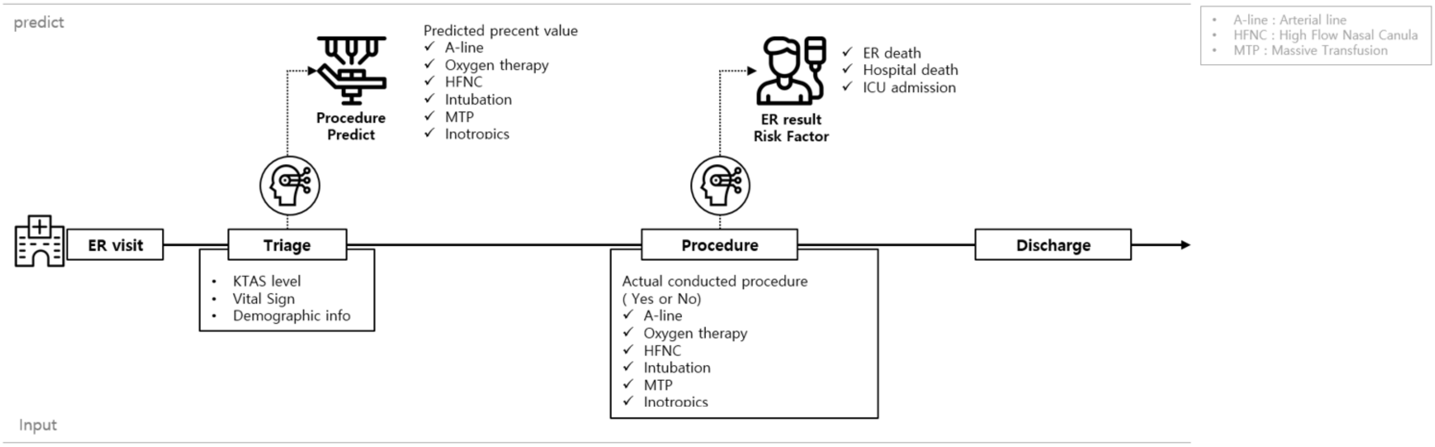

Prediction Model of Critical Interventions (CrIs) were bases on overall timeline of patient progression through the emergency department starting from the emergency room visit, to triage for the evaluation of the first assessment, execution of procedures, and discharge. *A-line* arterial line, *HFNC* high-flow nasal cannula, *MTP* massive transfusion protocol, *Inotropics* inotropic agents including epinephrine, norepinephrine, dopamine, dobutamine, and vasopressin.

Supplementary Figure. 4 Flowsheet used at the study center.

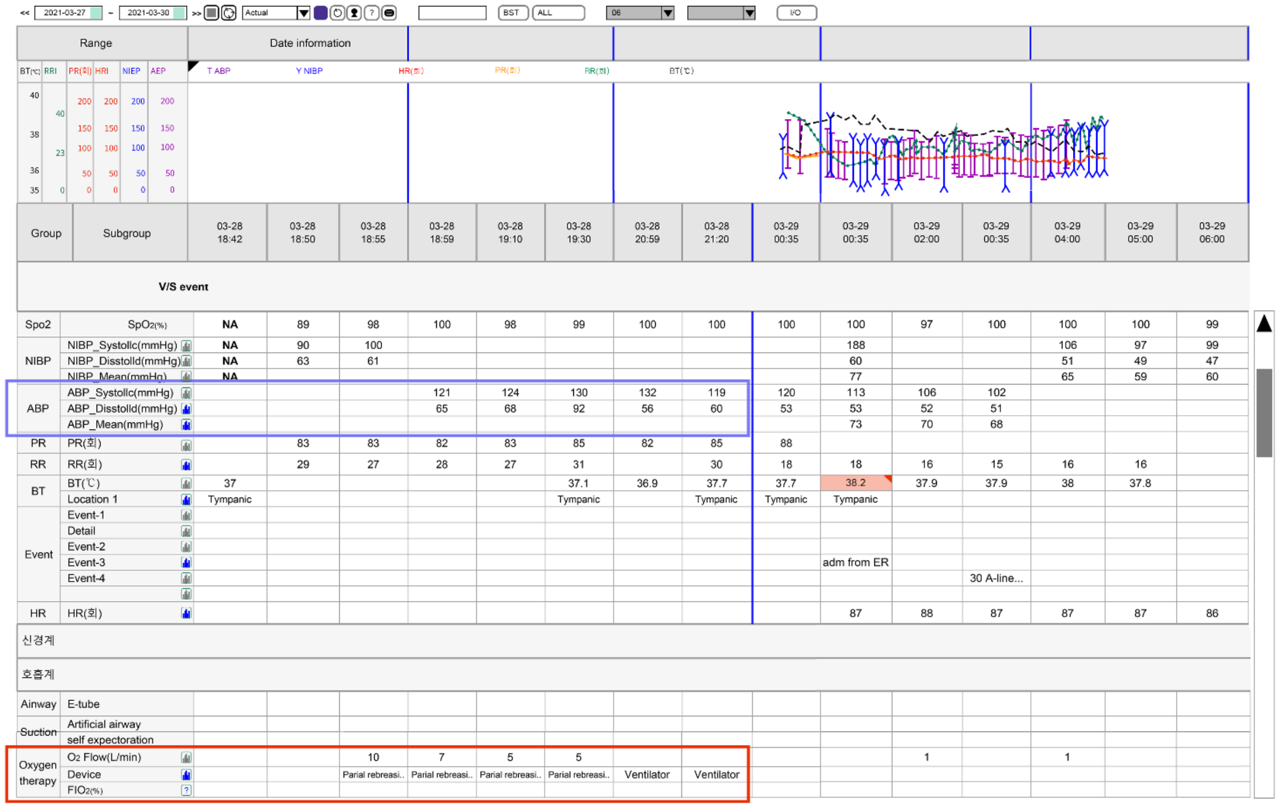

The flowsheet is an electronic medical record-integrated dashboard that summarizes the patient's current information and provides patient vital signs, mental status, and other important events. This information is stored and re-extracted from the clinical data warehouse. Boxes A and B indicate the information used in the present study. A. Arterial blood pressure is monitored and recorded in the flowsheet, with a record when an arterial line is inserted. B. Oxygen therapy-related information is also recorded, including the use of oxygen therapy devices such as nasal cannula and mask, high-flow nasal cannula, intubation and ventilator, and oxygen flow.

Supplementary Figure 5. EMR Integration process of CRI.

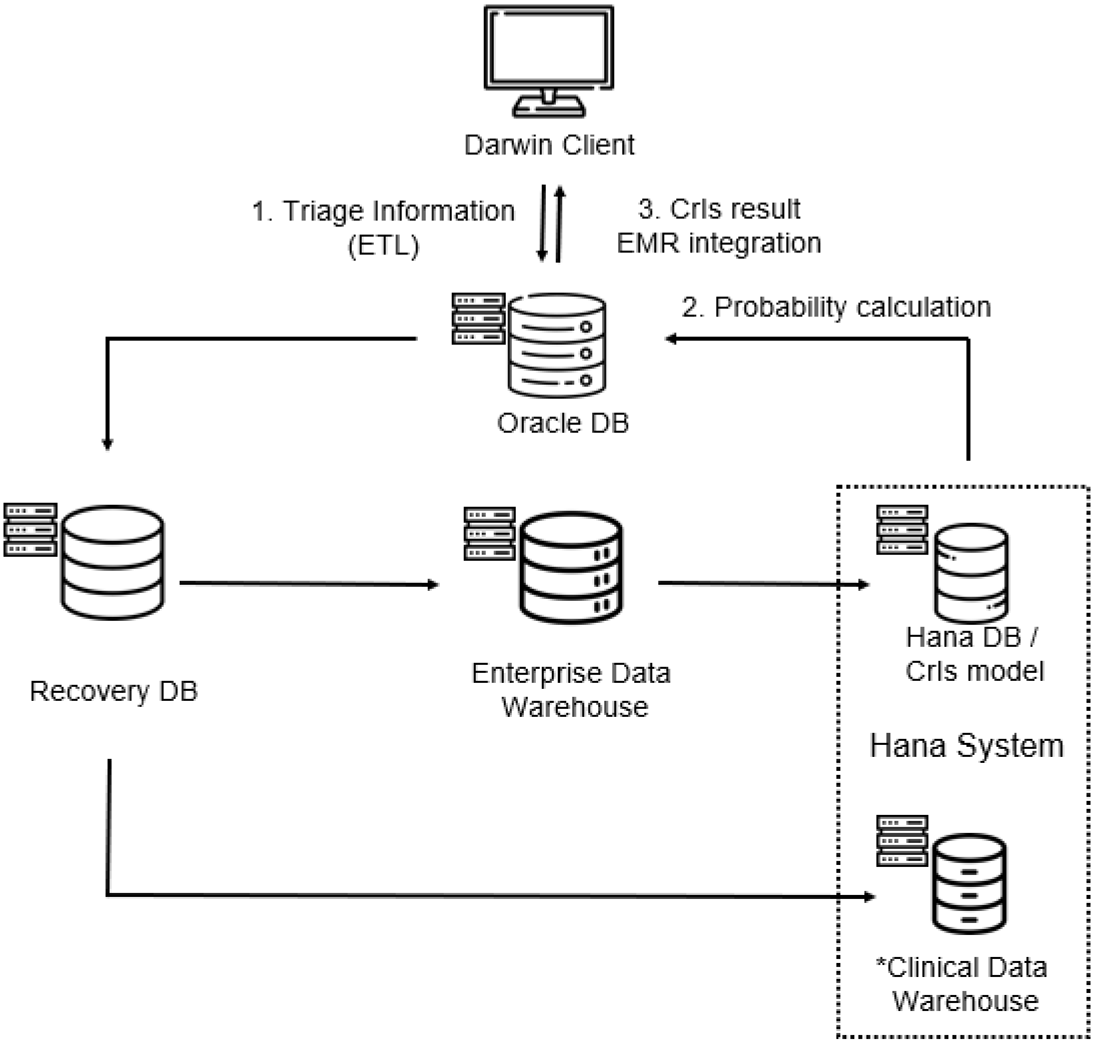

Initial triage inputs were collected in the EMR and transferred every 10 minutes to the HANA Database, which houses the CRIs models. Finally, each probability was computed and integrated into the EMR system once more. DARWIN: data analytics and research window for integrated knowledge ; EMR : Electronic medical record ; EDW : Enterprise data warehouse. ETL : Extraction, Transformation, and Loading ; CrIs : Critical Interventions.; HANA database : Hochleistungs ANalyse Anwendung database

Supplementary Table 1. The numbers (percentage) of the missing or uncheckable variables.

	n	Missing frequency (%)
Age	137,833	0 (0%)
Sex	137,833	0 (0%)
KTAS	137,833	0 (0%)
Consciousness	137,833	0 (0%)
Ambulance	137,833	0 (0%)
Direct Visit	137,833	0 (0%)
Vital signs		
Pulse, /min	137,833	1,192 (0.86%)
Respiration, /min	137,833	1,218 (0.88%)
SPo2, %	137,833	3,463 (2.51%)
DBP, mmpg	137,833	253 (0.18%)
SBP, mmpg	137,833	253 (0.18%)
Temperature, ℃	137,833	1,084 (0.78%)

KTAS : Korea Triage Acuity Scale, AVPU : Alert DBP : Diastolic blood pressure ; SBP : Systolic blood pressure.

Vital sign and demographic recording is mandatory at the study site, and all patients including minor patients should have them completed. Only when it is very urgent situation and patient do not have checkable vital signs, triage nurse can enter vital sign as 'uncheckable'.

Supplementary Table 2. Prediction results of AUROC and AUPRC with 95% confidence intervals according to the machine learning method and other input factors.

	Input	Metric	**Deep Learning**	**Logistic Regression**	**Random Forest**	**XGBoost**
A-line	A	AUROC	0.890 (0.881 – 0.900)	0.886 (0.876 – 0.896)	0.874 (0.864 – 0.884)	0.892 (0.882 – 0.902)
		AUPRC	0.097 (0.087 – 0.107)	0.105 (0.095 – 0.115)	0.088 (0.078 – 0.098)	0.091 (0.081 – 0.101)
	B	AUROC	0.889 (0.879 – 0.899)	0.886 (0.876 – 0.896)	0.872 (0.862 – 0.882)	0.891 (0.881 – 0.901)
		AUPRC	0.093 (0.083 – 0.103)	0.106 (0.096 – 0.116)	0.095 (0.085 – 0.105)	0.089 (0.079 – 0.099)
Oxygen therapy	A	AUROC	0.914 (0.904 – 0.924)	0.91 (0.900 – 0.920)	0.901 (0.891 – 0.911)	0.908 (0.898 – 0.918)
		AUPRC	0.540 (0.530 – 0.550)	0.529 (0.519 – 0.539)	0.52 (0.510 – 0.530)	0.531 (0.521 – 0.541)
	B	AUROC	0.916 (0.906 – 0.926)	0.912 (0.902 – 0.922)	0.9 (0.892 – 0.910)	0.915 (0.905 – 0.925)
		AUPRC	0.537 (0.527 – 0.547)	0.530 (0.521 – 0.542)	0.516 (0.506 – 0.526)	0.537 (0.527 – 0.547)
HFNC	A	AUROC	0.958 (0.948 – 0.968)	0.96 (0.952 – 0.973)	0.957 (0.947 – 0.967)	0.945 (0.935 – 0.955)
		AUPRC	0.122 (0.112 – 0.132)	0.121 (0.111 – 0.131)	0.131 (0.121 – 0.141)	0.106 (0.096 – 0.116)
	B	AUROC	0.963 (0.953 – 0.973)	0.959 (0.949 – 0.969)	0.949 (0.939 – 0.959)	0.943 (0.933 – 0.953)
		AUPRC	0.122 (0.112 – 0.132)	0.124 (0.114 – 0.134)	0.127 (0.117 – 0.137)	0.106 (0.096 – 0.116)
Intubation	A	AUROC	0.949 (0.939 – 0.959)	0.947 (0.937 – 0.957)	0.955 (0.945 – 0.965)	0.939 (0.929 – 0.949)
		AUPRC	0.209 (0.199 – 0.219)	0.221 (0.211 – 0.231)	0.226 (0.216 – 0.236)	0.174 (0.164 – 0.184)
	B	AUROC	0.947 (0.937 – 0.957)	0.953 (0.943 – 0.963)	0.951 (0.941 – 0.961)	0.936 (0.926 – 0.946)
		AUPRC	0.209 (0.199 – 0.219)	0.223 (0.213 – 0.233)	0.226 (0.216 – 0.236)	0.174 (0.164 – 0.184)
MTP	A	AUROC	0.916 (0.906 – 0.926)	0.975 (0.965 – 0.985)	0.976 (0.966 – 0.986)	0.953 (0.943 – 0.963)
		AUPRC	0.143 (0.133 – 0.153)	0.121 (0.111 – 0.131)	0.159 (0.149 – 0.169)	0.249 (0.239 – 0.259)
	B	AUROC	0.923 (0.913 – 0.933)	0.981 (0.971 – 0.991)	0.983 (0.973 – 0.993)	0.959 (0.949 – 0.969)
		AUPRC	0.147 (0.137 – 0.157)	0.119 (0.109 – 0.129)	0.166 (0.156 – 0.176)	0.252 (0.242 – 0.262)
Inotropics	A	AUROC	0.911 (0.901 – 0.923)	0.905 (0.895 – 0.915)	0.892 (0.881 – 0.903)	0.898 (0.888 – 0.908)
		AUPRC	0.428 (0.418 – 0.438)	0.401 (0.390 – 0.412)	0.382 (0.372 – 0.392)	0.411 (0.401 – 0.421)
	B	AUROC	0.913 (0.903 – 0.922)	0.904 (0.894 – 0.914)	0.899 (0.889 – 0.909)	0.901 (0.891 – 0.912)
		AUPRC	0.423 (0.413 – 0.433)	0.401 (0.391 – 0.411)	0.379 (0.369 – 0.389)	0.407 (0.397 – 0.417)

A: Original + presence of lab order; B: Original + presence of lab order + categorized lab test results with normal range.

XGBoost extreme gradient boosting, *AUROC* area under the receiver operating characteristic curve, *AUPRC* area under the precision-recall curve, *A-line* arterial line insertion, *MTP* massive transfusion protocol.

Supplementary Table 3. Considered and selected hyperparameters in Xgboost modeling.

Parameters	Search Space; best parameter
**Maximum tree depth**	6,7,8,11,12,13,15; 7
**Minimum data**	8,12,14,22,25; 12
**Gamma**	0.001~0.07; 0.004
**Subsampling ratio**	0.3~0.9; 0.4
**Number of predictors**	3,4,5,11,12; 5
**Learning rate**	0.001~0.003; 0.002
